# Supplementary material for: Ecological inference using data from accelerometers needs careful protocols
Source: Methods Ecol Evol. 2022 Feb 7;13(4):813–25. doi: 10.1111/2041-210X.13804 (PMC9303593; doi:10.1111/2041-210X.13804)
Supplement: Supplementary file 1 — Table S1 [file MEE3-13-813-s001.docx]

# Supplementary Information

*Table SI1. Comparison of accelerometer sensitivity between different models. There are a few manufacturers that supply MEMS accelerometers with acceleration ranges of 2, 4, 8, and 16* g*, while the sensitivity, the smallest detectable/measurable change, at different ranges can vary greatly. The table below shows a list of sensors possessing comparable acceleration ranges, the first two of which are used on the devices discussed in this paper, with others from well-known manufacturers, in some cases with far less sensitivity than the median. LSM303DLHC is the accelerometer-magnetometer chip built on to type 2 tags, while the LSM9DS1 is the chip built on to type 1 tags. Note that type 1 and type 2 tags have a substantially different sensitivity, far higher in LSM9DS1 accelerometers.*

| **Manufacturer** | **Sensor** | **±2 g** | **±4 g** | **±8 g** | **±16 g** |
| --- | --- | --- | --- | --- | --- |
| **ST** | **LSM303DLHC** | 1 | 2 | 4 | 12 |
| **ST** | **LSM9DS1** | 0.061 | 0.122 | 0.244 | 0.732 |
| **ST** | **MIS2DH** | 0.98 | 1.95 | 3.91 | 11.72 |
| **TDK** | **ICM-20948** | 0.061 | 0.122 | 0.244 | 0.488 |
| **TDK** | **IAM-20381** | 0.061 | 0.122 | 0.244 | 0.488 |
| **TDK** | **IIM-42652** | 0.061 | 0.122 | 0.244 | 0.488 |
|  |  |  |  |  |  |
| **Analog** | **ADXL346** | 0.015 | 7.81 | 15.63 | 31.25 |
| **Kionix** | **KX132-1211** | 0.061 | 0.122 | 0.244 | 0.488 |
| **Bosch Sensortec** | **BMA180** | 0.244 | 0.488 | 0.977 | 1.953 |
| **Bosch Sensortec** | **BMA456** | 0.061 | 0.122 | 0.244 | 0.488 |
|  | ***Median*** | 0.061 | 0.122 | 0.244 | 0.488 |
|  |  |  |  |  | *mg/LSB* |
